# Mechanical stress influences the morphology and function of human uterosacral ligament fibroblasts and activates the p38 MAPK pathway

**DOI:** 10.1007/s00192-021-04850-7

**Published:** 2021-05-25

**Authors:** Yapei Zhu, Lei Li, Ting Xie, Tao Guo, Lan Zhu, Zhijing Sun

**Affiliations:** 1 Department of Obstetrics and Gynecology, Peking Union Medical College Hospital, Chinese Academy of Medical Sciences & Peking Union Medical College, Beijing, China; 2 National Clinical Research Center for Obstetric & Gynecologic Disease, Beijing, China; 3 Department of Medical Research Center, Peking Union Medical College Hospital, Chinese Academy of Medical Sciences & Peking Union Medical College, Beijing, China; 4 Department of Obstetrics and Gynecology, Peking Union Medical College Hospital, 1 Shuaifuyuan, Dongcheng District Beijing, 100730 People’s Republic of China

**Keywords:** Pelvic organ prolapse, Human uterosacral ligament fibroblasts, Mechanical stress, p38 MAPK pathway

## Abstract

**Introduction and hypothesis:**

Pelvic organ prolapse (POP) is a common condition in older women that affects quality of life. Mechanical injury of the pelvic floor support system contributes to POP development. In our study, we aimed to examine the mechanical damage to human uterosacral ligament fibroblasts (hUSLFs) to preliminarily explore the mechanism of mechanical transduction in POP.

**Methods:**

hUSLFs were derived from POP and non-POP patients. Mechanical stress was induced by the FX-5000 T-cell stress loading system. Student’s *t*-test was used for comparisons between different groups.

**Results:**

We found that hUSLFs from POP patients were larger and longer than those from non-POP patients and exhibited cytoskeleton F-actin rearrangement. Collagen I and III expression levels were lower and matrix metalloproteinase 1 (MMP1) levels were higher in POP patients than in non-POP patients. Additionally, the apoptosis rate was significantly increased in POP patients compared to non-POP patients. After mechanical stretching, hUSLFs underwent a POP-like transformation. Cells became longer, and the cytoskeleton became thicker and rearranged. The extracellular matrix (ECM) was remodelled because of the upregulation of collagen I and III expression and downregulation of MMP1 expression. Mechanical stress also induced hUSLF apoptosis. Notably, we found that the p38 MAPK pathway was activated by mechanical stretching.

**Conclusions:**

Mechanical stress induced morphological changes in ligament fibroblasts, leading to cytoskeleton and ECM remodelling and cell apoptosis. p38 MAPK might be involved in this process, providing novel insights into the mechanical biology of and possible therapies for this disease.

## Introduction

Pelvic organ prolapse (POP) is a female pelvic floor disorder involving pelvic organ protrusion caused by the damage and degeneration of the supportive structures of the pelvic floor. The main symptoms are prolapse of the vagina and/or uterus, with or without urinary incontinence and defecation and sexual dysfunction [1]. The incidence of POP is approximately 25% in Chinese women over the age of 60 years, with 43%–76% of patients requiring surgical treatment [2]. POP has a negative impact on the quality of women’s lives. Increasing attention has been paid to this disorder due to the ageing of the population and the “two-child” policy. Childbirth, pregnancy and long-term constipation are well-known to be the main risk factors for POP [3, 4], suggesting that the occurrence of POP is closely related to mechanical injury of the pelvic floor. Mechanobiology and mechanotherapy are currently hot research topics that have important scientific significance in disease regulation, signal transmission and biological treatment. Therefore, studying the mechanism of POP and finding new therapeutic methods from the perspective of mechanical biology and mechanical therapeutics are promising. However, further exploration on pelvic floor disorders is needed [5, 6].

Previous studies [7] have demonstrated that injury to pelvic floor supportive structures, especially connective tissue ligaments, is the main contributor to the development of POP. Connective tissue is mainly composed of fibroblasts and the various extracellular matrix (ECM) components between them, which can connect the supporting tissues of the pelvic floor and transmit and disperse external forces. When abdominal pressure increases, such as during pregnancy, childbirth or constipation, the pelvic floor tissues are subjected to extra tensile force. Fibroblasts in the connective tissues respond to mechanical stress and convert these mechanical signals into intracellular biochemical signals. This mechanical signal transduction pathway leads to alterations in gene expression and cellular activities [8, 9].

Studies [10–13] have shown that disorder of mechanical homeostasis is related to numerous diseases. POP also results from pelvic floor supporting tissues being pulled by an external force for an extended period, which disturbs the mechanical balance of the pelvic floor. Mechanical injury of the pelvic floor may disrupt these supportive tissues and connections through remodelling of the ECM. Gong et al. [14] reported that strong or long-term mechanical stress induced an imbalance between matrix metalloproteinases (MMPs), which can degrade ECM components, and their specific tissue inhibitors of metalloproteinases (TIMPs), leading to abnormalities in collagen anabolism. Furthermore, these changes were involved in several intercellular signalling pathways, including the TGF-β/Smad, PI3K/AKT and NF-κB pathways. Ming et al. [15] revealed that mechanical stress inhibited cell proliferation and increased cell senescence. The authors mainly focused on the effects of excessive mechanical force and hydrogen peroxide, which could induce ECM remodelling via the TGF-β1 signalling pathway.

Our previous gene microarray analysis showed that the MAPK pathway was abnormally expressed in patients with POP [16]. The MAPK signalling pathway is a well-recognized pathway with activities related to mechanical transduction and cell apoptosis. Previous research [17] has demonstrated that the p38 MAPK pathway was activated by various mechanical stimuli and was transduced through the focal adhesion kinase pathway downstream of transmembrane integrins. Another study [18] found that in periodontal ligament fibroblasts, the p38 MAPK pathway was activated upon cyclic deformation and tensile stress and that cell proliferation was inhibited through this pathway.

Research on the mechanism of pelvic floor tissue conduction induced by mechanical force is limited, and the changes that occur in ECM components are controversial. Therefore, in our study, to further clarify the role of mechanical stress in the occurrence and development of POP, we compared how human uterosacral ligament fibroblasts (hUSLFs) from POP and non-POP patients respond to a stress state. We hypothesised that the MAPK pathway might be involved in mechanical trauma-induced POP, providing new insights into the pathophysiology of POP.

## Materials and methods

### Subjects

In this study, uterosacral ligament tissues were obtained from eight women with POP and ten women without prolapse at Peking Union Medical College Hospital (PUMCH). POP patients were diagnosed as having stage III or IV POP according to the Pelvic Organ Prolapse Quantification (POP-Q) System and underwent total vaginal hysterectomy, while non-POP patients were women who suffered from benign gynaecological diseases (such as cervical intra-epithelial neoplasia and non-functional ovarian benign cysts) and underwent laparoscopic-assisted vaginal hysterectomy. This study was approved by the ethics committee of PUMCH. All the participants signed an informed consent form.

We excluded women with urinary incontinence; uterine leiomyoma, adenomyosis, endometriosis or other oestrogen-related diseases; chronic pelvic inflammation; malignant tumours; or collagen deficiency syndrome and those who underwent previous pelvic surgery. Sociological and basic information, including age, height, body weight and history of pregnancy and childbirth, was collected.

### Cell culture

Approximately 0.5- to 1-cm pieces of uterosacral ligament tissues were collected from the posterior attachment to the cervix during surgery. The tissues were immediately placed in Dulbecco’s modified Eagle’s medium (DMEM; Gibco, Carlsbad, CA, USA), washed with phosphate-buffered saline (PBS) containing 100 U/ml penicillin and 100 mg/ml streptomycin (Gibco), and then cut into small pieces with sterile ophthalmic scissors. The tissues were digested with 10 mg/ml collagenase I (Sigma, St. Louis, MO, USA) for 3 h at 37 °C in 5% CO_2_, and digestion was terminated with foetal bovine serum (FBS; Gibco). The digestion liquid was filtered through a 200-mesh screen (75-μm pore size) and then centrifuged at 800 rpm for 5 min. The supernatant was discarded, and the cells were suspended in DMEM containing 10% FBS and cultured in a 25-cm^2^ culture flask. The culture medium was changed every 2 days, and primary fibroblasts were grown to spread across the culture medium for passage. Fibroblasts were used at passage 3–5. The cells were observed with an Olympus FV500 optical microscope (Olympus, Tokyo, Japan).

### Immunocytochemistry

To stain fibroblast-related proteins, the cells were permeabilized with 0.1% Triton X-100 and then incubation with the following primary antibodies overnight at 4 °C: anti-vimentin (1:200, ZSGB-Bio, Beijing, China), anti-fibroblast specific protein 1 (FSP-1) (1:200, ZSGB-Bio), anti-α-smooth muscle actin (α-SMA) (1:200, ZSGB-Bio) and anti-cell keratin 5/6 (CK5/6) (1:200, ZSGB-Bio). The cells were incubated with biotinylated secondary antibodies (1:4000, ZSGB-Bio) for 2 h at room temperature. 3,3-Diaminobenzidine (DAB) solution (ZSGB-Bio) was then used to visualize the localization of the target proteins. Finally, the cells were counterstained with haematoxylin (ZSGB-Bio) for nuclear staining. PBS was used as the negative control. The slides were observed with an Olympus FV500 optical microscope (Olympus, Tokyo, Japan).

### Mechanical stress

A cell suspension containing hUSLFs (2 × 10^5^) was seeded on a UniFlex Culture Plate-Collagen Type I (Flexcell, McKeesport, PA, USA), a 6-well plate that had an elastic basement membrane pretreated with type I collagen and incubated for 24 h. After the cells adhered, the cell plate was placed onto the strain loading plate of the FX-5000 T instrument (Flexcell) under a loading strain of 1/2 sin waveform uniaxial cyclic stress loading, a tensile strain of 10% tensile strain and a frequency of 0.1 Hz. The experiments were repeated three times.

### Quantitative real-time polymerase chain reaction (qPCR)

Total RNA was extracted from hUSLFs using TRIzol reagent (Promega, Madison, WI, USA) and cDNA was subsequently synthesized using a reverse transcription kit (Takara, Tokyo, Japan) according to the manufacturer’s instructions. qPCR was performed using the SYBR Green Real-time PCR system (Promega). Human GAPDH was amplified as an internal control. qPCR was performed as follows: 2 min at 95 °C; 40 cycles of 3 s at 95 °C and 30 s at 60 °C; and 60–95 °C for the dissociation curve. The data were analysed using the 2^-ΔΔCq^ method relative to GAPDH. The primer sequence information is as follows: *bax*, forward-GTCTTTTTCCGAGTGGCAGC, reverse-GTCCAATGTCCAGCCCATGA; *bad*, forward-ATGGTCACCTTACCTCTGCAA, reverse-TCATAGCGTCGGTTGATGTCG; and *GAPDH*, forward-AACGTGTCAGTGGTGGACCTG, reverse-GAGACCACCTGGTGCTCAGTG.

### Western blot analysis

hUSLFs were harvested, washed in cold PBS and then lysed on ice for 30 min in lysis buffer (Beyotime, Shanghai, China). The extracts were centrifuged at 12,000 rpm at 4 °C for 15 min, and the supernatants were carefully collected as total cell protein extracts. The protein concentration was detected by the bicinchoninic acid protein assay kit (Beyotime) according to the instructions. A total of 30 μg of protein was added to each lane of a Tris-glycine gel and separated by 10% SDS-PAGE. After electrophoresis, the proteins were transferred to a polyvinylidene difluoride membrane. The membrane was incubated with the following primary antibodies overnight at 4 °C: anti-p38 (1:1000, Cell Signalling Technology, Boston, MA, USA), anti-p-p38 (1:1000, Cell Signalling Technology), anti-extracellular signal-regulated kinase (ERK) (1:1000, Cell Signalling Technology), anti-p-ERK (1:1000, Cell Signalling Technology), anti-c-jun N-terminal kinase (JNK) (1:1000, Cell Signalling Technology), anti-p-JNK (1:1000, Cell Signalling Technology) and anti-α-tubulin (1:2000, Abcam, Cambridge, UK). After being washed in TBST three times, the membrane was incubated with horseradish peroxidase-conjugated secondary antibodies (Cell Signalling Technology) at 37 °C for 2 h. Rapid Step ECL Reagent (Millipore, Schwalbach, Germany) was used to visualize the proteins. All the experiments were performed at least three times.

### Cell apoptosis analysis by FACS

Cell apoptosis assay was examined using an Annexin V-FITC/PI Apoptosis kit according to the manufacturer’s protocol (ThermoFisher, Waltham, MA, USA). Briefly, cells were digested with trypsin (Gibco) and washed twice in cold PBS. The cells were then centrifuged at 1500 r/min for 5 min. The supernatant was removed, and the precipitate was resuspended in 1 × annexin-binding buffer at a density of 1.0 × 10^6^cells/ml. A total of 100 μl of the solution was incubated with 5 μl of FITC annexin V and 1 μl of PI (100 μg/ml) for 15 min in the dark. After 400 μl of 1× binding buffer was added to each sample tube, the cell samples were detected with an Accuri C6 instrument (BD, Franklin Lakes, NJ, USA). The data were analysed with FlowJo software.

### Immunofluorescence staining

hUSLFs were seeded in a 24-well plate at the density of 2.4 × 10^5^ cells/ml and cultured in 5% CO_2_ at 37 °C. Paraformaldehyde in PBS (Wuhan Servicebio Technology Co., Ltd., Wuhan, China) was used to fix the fibroblasts. To stain cytoskeleton F-actin, the cells were permeabilized with 0.1% TritonX-100 (Solarbio, Beijing, China) for 5 min, treated with phalloidin (5 μg/ml) (Wuhan Servicebio Technology Co., Ltd.) and incubated for 60 min at room temperature in the dark. DAPI (5 μg/ml, 100 μl/well) (Solarbio) was used to stain the nuclei for 10 min in the dark. The cell cytoskeleton was observed using a confocal laser scanning microscope (Ti2-E/A1R+; Nikon, Tokyo, Japan).

### Statistical analysis

Statistical analyses were conducted with SPSS 19.0 software (IBM SPSS, Armonk, NY, USA). The clinical data of the recruited women are presented as the mean ± standard deviation (SD) or median ± range. All the experiments were repeated at least three times. Student’s *t*-test or paired *t*-test was used for comparisons between POP and non-POP patients and comparisons between fibroblasts with and without mechanical stretching. *P* < 0.05 was considered significant.

## Results

### Characteristics of the women recruited for this study

In this study, we recruited eight women with POP of POP-Q stages III and IV and ten women without prolapse. Table 1 shows the clinical characteristics of the women with and without POP. No significant differences in age, body mass index, menopausal status, gravidity or vaginal parity were observed between the two groups, indicating these characteristics were matched between the two groups.

**Table 1:** Clinical characteristics of the enrolled women

Characteristic	Control (*n* = 10)	POP (*n* = 8)	*P*
Age(years, mean ± SD)	55.60 ± 4.90	58.88 ± 8.34	0.313
BMI(kg/m^2^, mean ± SD)	23.43 ± 2.23	24.29 ± 2.56	0.458
Menopause (*n*, %)	8 (80.0%)	7 (87.5%)	1.000
Gravidity (median, range)	3 (1–3)	1 (1–1)	0.440
Vaginal delivery (median, range)	3 (2–3)	2 (1–2)	0.069

POP: pelvic organ prolapse; BMI: body mass index

### Comparison of the morphology of hUSLFs from the non-POP and POP groups

**Fig. 1:**
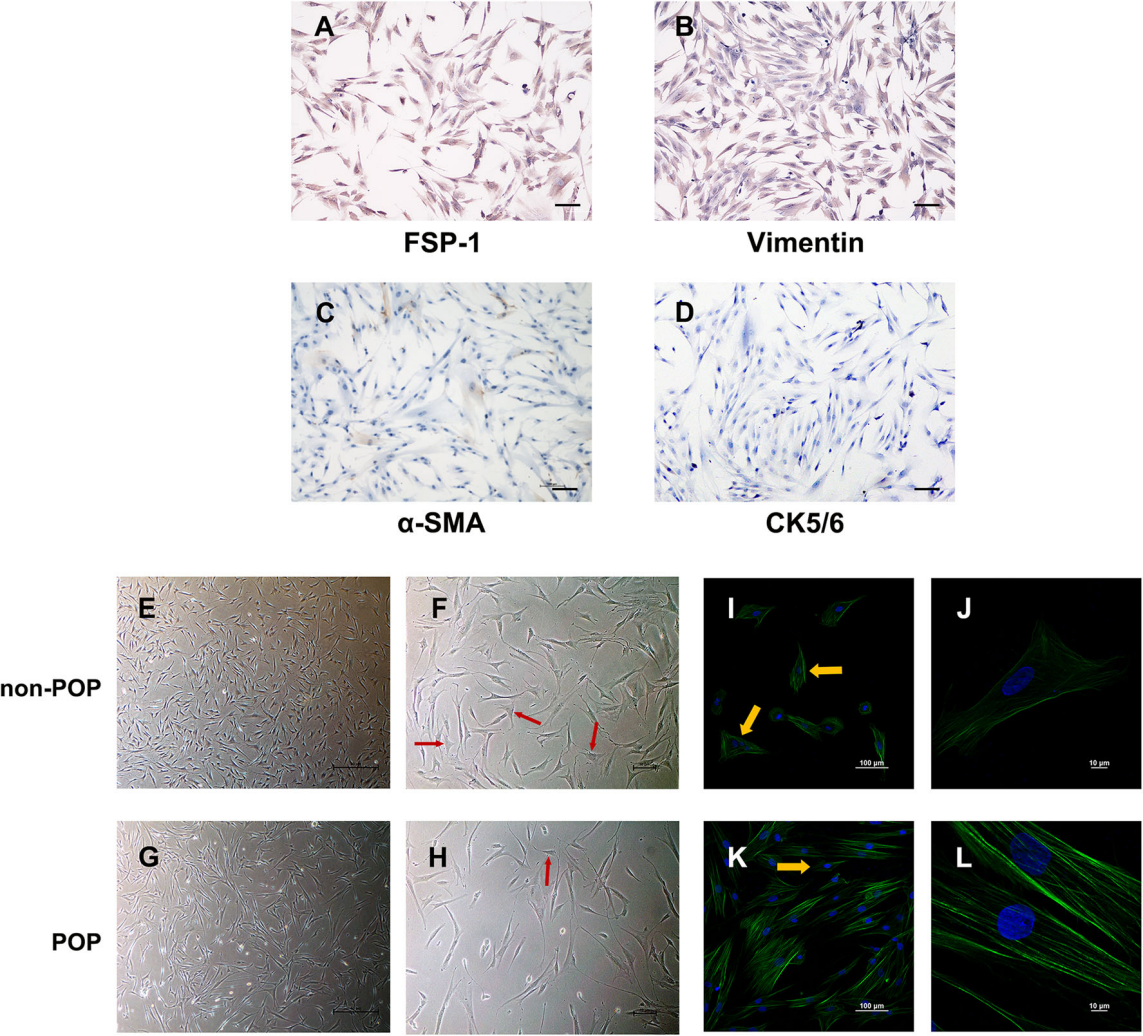
Comparison of the morphology of hUSLFs from the non-POP and POP groups. Identification of hUSLFs by immunocytochemical staining for (A) FSP-1, (B) vimentin, (C) α-SMA and (D) CK5/6 (magnification: ×100; scale bar = 10 μm). E–G: Bright-field images of hUSLFs observed at low power (E and G, magnification: ×40; scale bar = 100 μm) and high power (F and H, magnification: ×100; scale bar = 10 μm). The red arrows indicate triangular or polygonal cells. I–L: Images of the hUSLF cytoskeleton by immunofluorescence staining for F-actin (green) and DAPI staining for nuclei (blue). The cells were observed under a confocal laser scanning microscope at low power (I and K, magnification: ×200; scale bar = 100 μm) and high power (J and L, magnification: ×1000; scale bar = 10 μm). The yellow arrows indicate triangular or polygonal cells

We cultured primary cells derived from the uterosacral ligament tissues of women with and without POP and identified the cells by immunocytochemical staining. As shown in Fig. 1, the cultured primary cells from both POP and non-POP patients were negative for α-SMA and CK5/6 but positive for FSP-1 and vimentin (Fig. 1A, B, C, D). Therefore, the cultured primary cells were human uterosacral ligament fibroblasts (hUSLFs) and not smooth muscle cells or epithelial cells. The purity of the hUSLFs was > 90%.

Additionally, we observed that most hUSLFs from both the POP and non-POP groups were spindle shaped and that a few hUSLFs were triangular or polygonal after 3–5 passages (Fig. 1E, F, G, H). When hUSLFs spread over the bottom of the culture dish, they were tightly connected to each other and arranged in a bundled mess. Furthermore, it was observed that the hUSLFs from the POP group were generally longer and that triangular or polygonal cells were less common in the POP group than in the non-POP group.

To further observe the differences in the cell morphology and the cytoskeleton between the two groups, FITC-labelled phalloidin was used to label the F-actin fibrils of the cytoskeleton. We found that F-actin appeared more well defined, exhibited brighter green fluorescence and presented as more discrete filamentsin in the POP group than in the non-POP group. The F-actin stress fibres in the POP group showed a single direction, were parallel to the long axis of the cells and were significantly thickened (Fig. 1I, J, K, L). We also observed that the hUSLFs from the non-POP group were shorter and wider than those from the POP group and were commonly triangular or polygonal; however, the hUSLFs in the POP group were slender, exhibited a larger cell volume and were rarely triangular or polygonal. These finding are consistent with the above-mentioned optical microscopy observations.

### Comparison of ECM-related protein expression in hUSLFs from the non-POP and POP groups

**Fig. 2:**
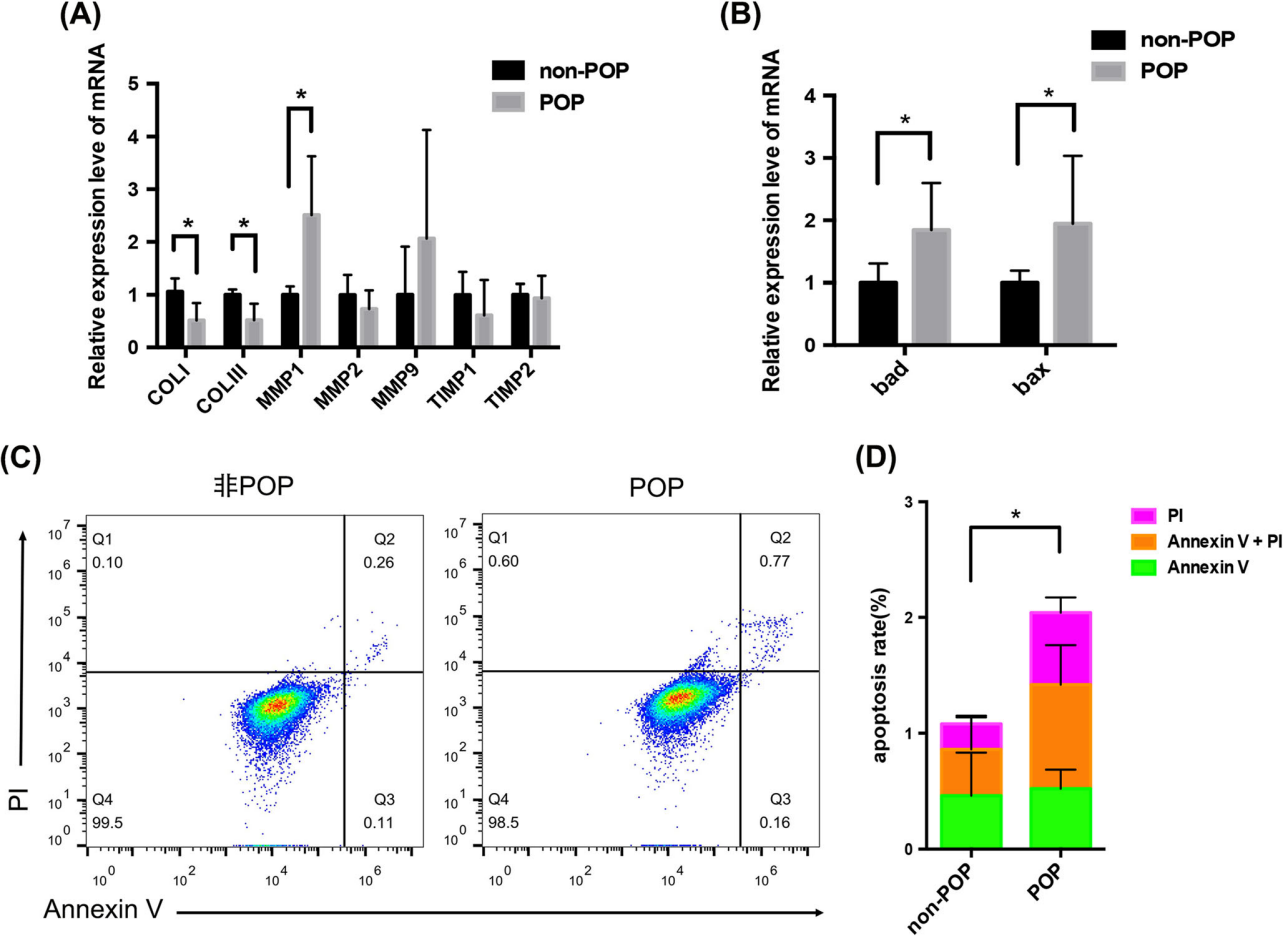
Comparison of ECM-related protein expression in and apoptosis of hUSLFs from the non-POP and POP groups. (A) The levels of the ECM-related proteins collagen I, collagen III, MMP1, MMP2, MMP9, TIMP1 and TIMP2 in hUSLFs from the non-POP and POP groups were examined by qPCR. (B) The levels of the apoptosis-related proteins *bad* and *bax* in hUSLFs from the non-POP and POP groups were examined by qPCR. (C) Cells from the non-POP and POP groups were stained with an Annexin V-FITC/PI staining kit and analysed by flow cytometry. (D) Statistical analysis of the apoptosis rate. Unpaired *t*-tests were performed. The data are presented as the mean ± SD. *P < 0.05

In our study, we examined the expression of the ECM-related genes in cultured hUSLFs from the two groups. As shown in Fig. 2A, the mRNA levels of collagen I and III in the POP group were significantly lower than those in the non-POP group (*P* = 0.034, *P* = 0.035, respectively); however, the mRNA levels of MMP1 were increased in the POP group compared to the non-POP group, and the difference was borderline significant (*P* = 0.054). Meanwhile, the mRNA level of MMP2 in the POP group was slightly lower than that in the non-POP group, and the MMP9 level was higher in the POP group. However, the differences were not significant. The mRNA levels of TIMP1 and TIMP2 were not changed between the two groups. These results suggest that ECM synthesis and degradation were altered in hUSLFs.

### Comparison of hUSLF apoptosis between the non-POP and POP groups

To observe the difference in hUSLF apoptosis between the POP and non-POP groups, we performed qPCR and flow cytometry to detect the expression of the apoptosis-related genes *bad* and *bax* in hUSLFs between the two groups. The mRNA levels of *bad* and *bax* in the POP group were higher than those in the non-POP group, and the differences were statistically significant (*P* = 0.049 and*P* = 0.028, respectively) (Fig. 2B). Additionally, annexin V-FITC/PI staining showed that, compared to that of the non-POP group, the apoptosis rate of the POP group was significantly increased (*P* = 0.045) (Fig. 2C, D).

### Mechanical stretching altered the morphology of hUSLFs and induced cytoskeleton remodelling

**Fig. 3:**
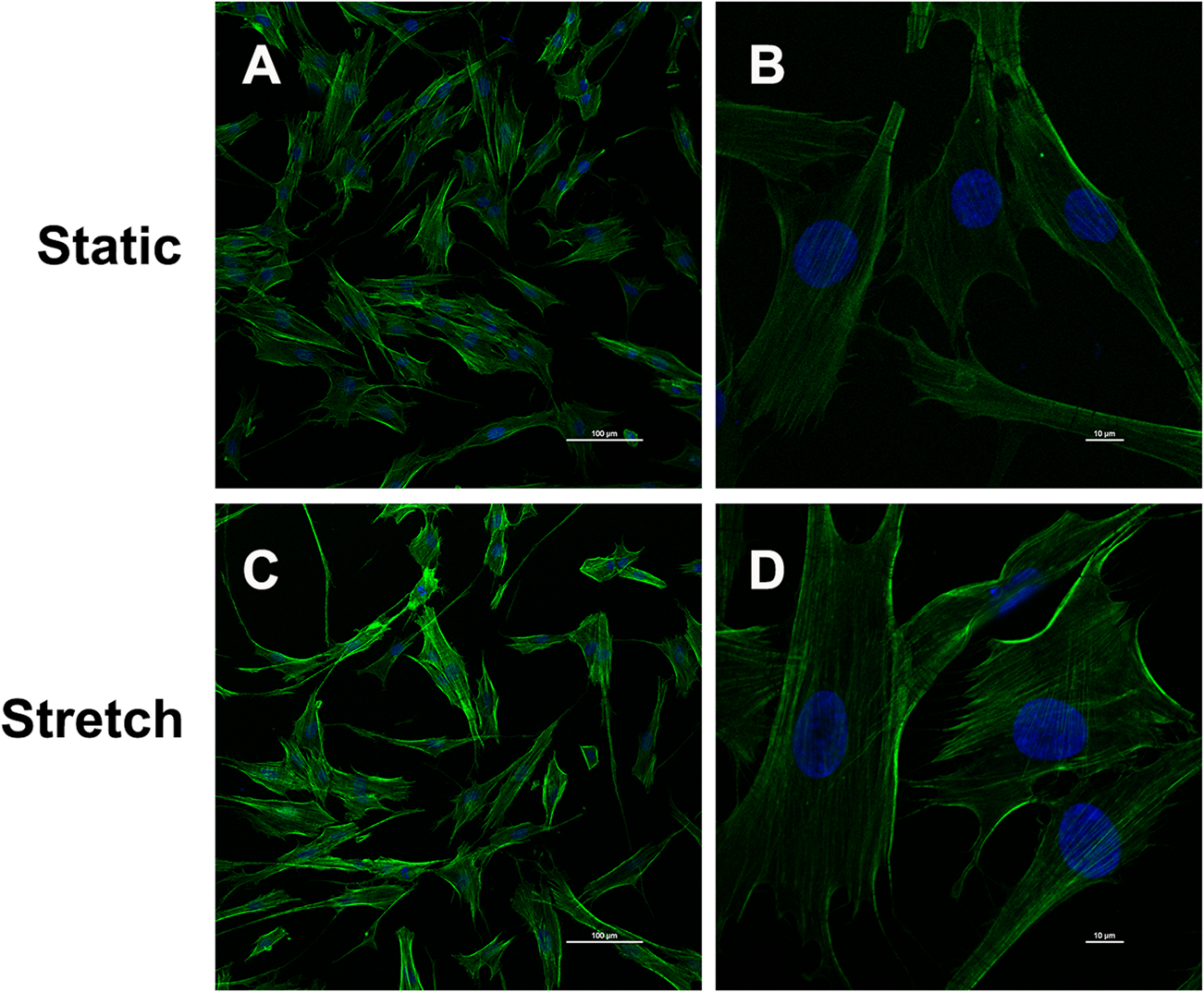
Mechanical stretching altered the morphology of hUSLFs and induced cytoskeleton remodelling. hUSLFs from the non-POP group were exposed to tension stress (0.1-Hz uniaxial, 10% elongation) for 24 h and then subjected to immunofluorescence staining for F-actin (green) and DAPI staining for nuclei (blue). The cells were observed under a confocal laser scanning microscope at low power (A and C, magnification: ×200; scale bar = 100 μm) and high power (B and D, magnification: ×1000; scale bar = 10 μm)

For further investigation, we simulated the effects of mechanical stretching on POP in hUSLFs in vitro. Changes in cell morphology and cytoskeleton were observed in hUSLFs from the non-POP group before and after tension stress (0.1-Hz uniaxial, 10% elongation) for 24 h (Fig. 3). Compared with the static cultured hUSLFs (Fig. 3A, B), the uniaxially stretched hUSLFs exhibited a larger cell volume and were more slender (Fig. 3C, D). F-actin stress fibres in the uniaxially stretched hUSLFs also showed a single direction, were parallel to the long axis of the cells and were significantly thickened, suggesting that they were quite similar to the static cultured hUSLFs in the POP group, as shown in Fig. 1.This indicates that mechanical stretching might cause normal cells to adopt a POP-like state.

### Mechanical stretching induced ECM remodelling and apoptosis of hUSLFs

**Fig. 4:**
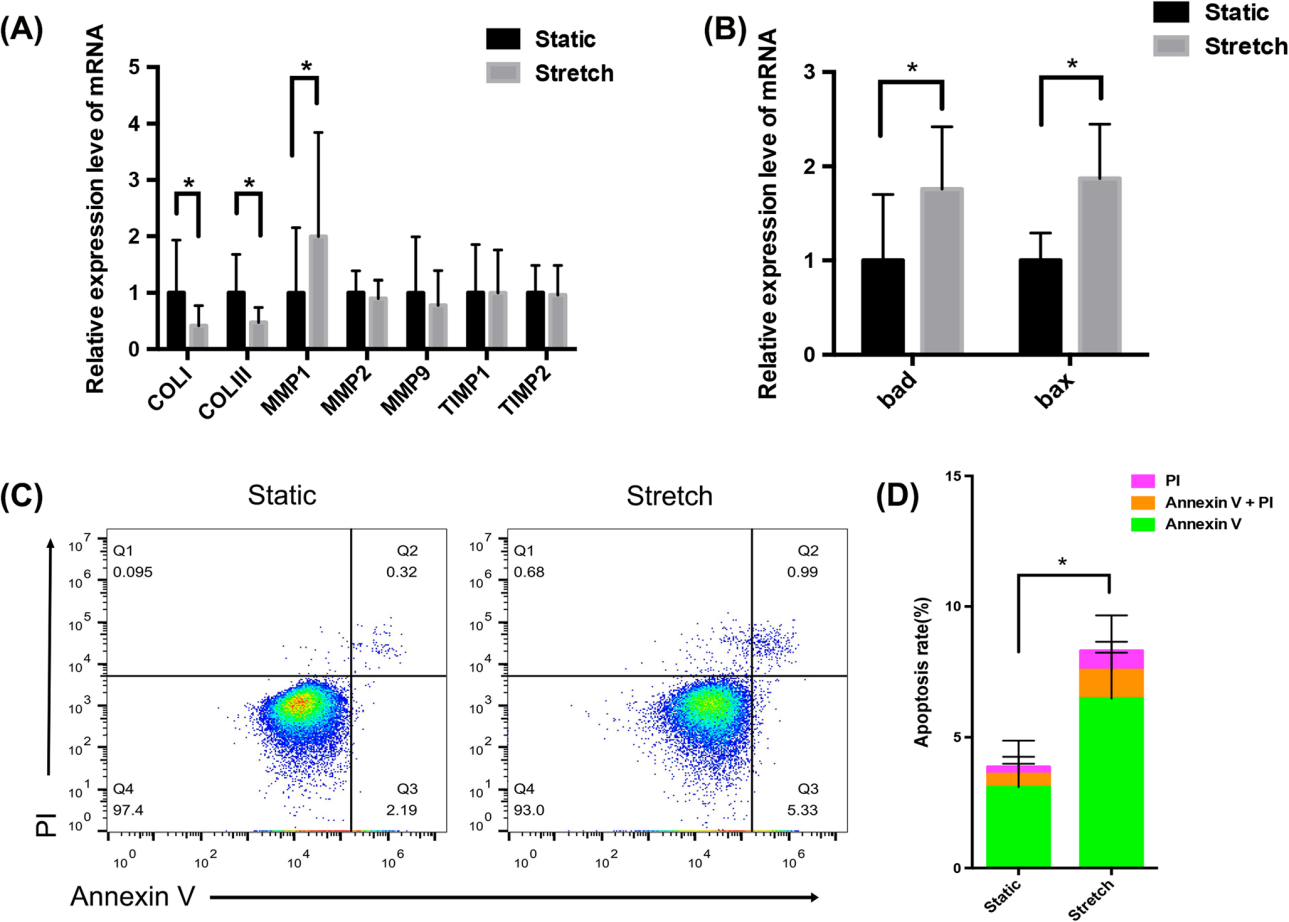
Mechanical stretching induced ECM remodelling and apoptosis of hUSLFs. hUSLFs from the non-POP group were exposed to tension stress (0.1-Hz uniaxial, 10% elongation) for 24 h and then subjected to qPCR and flow cytometry. (A) The levels of the ECM-related proteins collagen I, collagen III, MMP1, MMP2, MMP9, TIMP1 and TIMP2 in the static and stretched cells were examined by qPCR. (B) The levels of the apoptosis-related protein *bad* and *bax* in static and stretched cells were examined by qPCR. (C) Cells in the static and stretched groups were stained with an annexin V-FITC/PI staining kit and analysed by flow cytometry. (D) Statistical analysis of the apoptosis rate. Unpaired *t*-tests were performed. The data are presented as the mean ± SD. *P < 0.05

To examine the effects of mechanical stress on ECM-related proteins in hUSLFs, the expression levels of ECM-related proteins were detected before and after mechanical stress. As displayed in Fig. 4A, the mRNA levels of collagen I and III were significantly decreased after stretching (*P* = 0.034 and *P* = 0.039, respectively). In contrast, the mRNA level of MMP1 was notably increased after stretching (*P* = 0.042). Meanwhile, the mRNA levels of MMP2, MMP9, TIMP1 and TIMP2 were not obviously different before and after stress. Interestingly, the alterations in these ECM-related proteins in hUSLFs after stretching were consistent with the differences between the non-POP group and POP group reported above.

In addition, the expression of the apoptosis-related genes *bad* and *bax* was increased in hUSLFs after tension stress. The differences in the levels of both proteins were statistically significant (*P* = 0.019 and *P* = 0.034, respectively) (Fig. 4B). To further verify that the cells underwent apoptosis, apoptotic cells were detected by flow cytometry after staining with annexin V-FITC/PI. Compared to that of the static cultured hUSLFs, the apoptosis rate of hUSLFs that underwent mechanism stress was significantly increased (*P* = 0.045) (Fig. 4C, D).

### Mechanical stretching activated the p38 MAPK pathway in hUSLFs

**Fig. 5:**
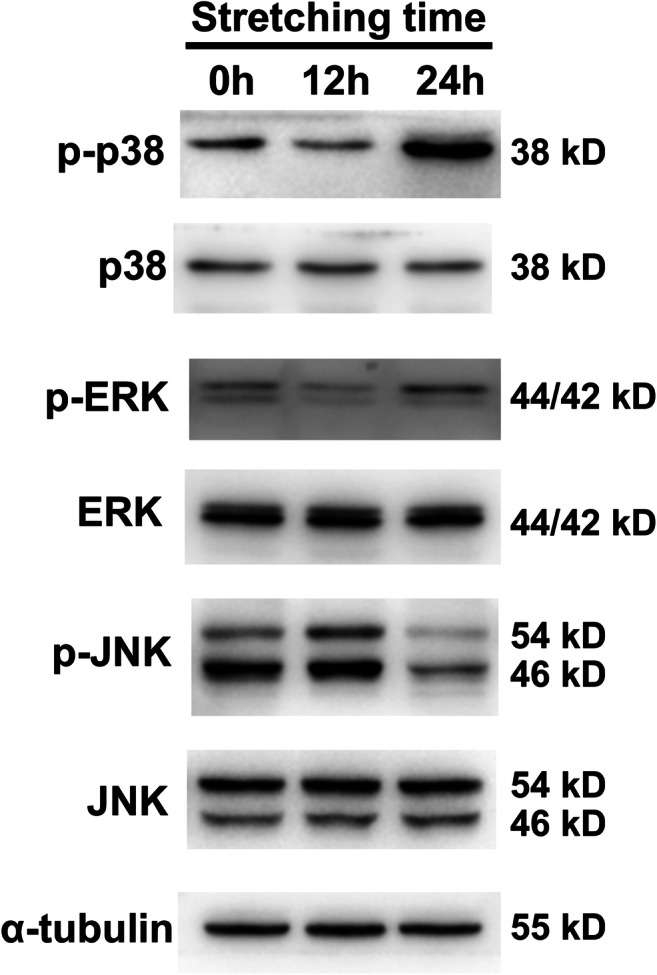
Mechanical stretching activated the MAPK-p38 pathway in hUSLFs. hUSLFs from the non-POP group were exposed to tension stress (0.1-Hz uniaxial, 10% elongation) for 12 or 24 h, and then p38, ERK and JNK expression and the phosphorylation of these proteins were assessed by Western blotting

To further explore the underlying mechanism of the effects of mechanical stretching on hUSLFs, we detected the phosphorylation levels of MAPK family proteins (p38, ERK and JNK) before and after stress. As shown in Fig. 5, there were no significant changes in the protein levels of p38, ERK or JNK before and after stretching. The protein levels of p-p38, p-ERK and p-JNK were not significantly changed after 12 h of stretching. However, the p-p38 expression level was significantly increased after 24 h of stretching. This result suggests that the p38-MAPK pathway was activated in hUSLFs after 24 h of stretching and that the p38-MAPK pathway might be involved in the regulation of the morphology and physiological function of hUSLFs induced by mechanical stretching.

## Discussion

The uterosacral ligament is the main ligament that maintains the normal position of the uterus in the female pelvic floor supportive system and consists of fibroblasts and ECM. Defects in the mechanical properties of the human uterosacral ligament may lead to the development of POP. Therefore, in this study, we cultured human uterosacral ligament fibroblasts in vitro as an experimental model and simulated the action of mechanical stretching on fibroblasts.

We first compared the morphology and function of primary cultured hUSLFs extracted from the uterosacral ligament tissues of POP and non-POP patients. We found that hUSLFs from the POP group were larger than those from the non-POP group and that the cells were elongated, and F-actin stress fibres became thicker. These results indicated that the cytoskeleton of hUSLFs changed during the development of POP, thus affecting cell morphology. F-actin stress fibres became thickened in cells, which may have caused a sub-tension state in the cells, resulting in relative weakening of cell contractility and responses to the external stimuli. Additionally, the apoptosis rate of hUSLFs from the POP group was significantly increased compared to that of hUSLFs from the non-POP group, which was consistent with previous studies [19, 20]. Wen et al. [19] and Saatli et al. [20] revealed that *bad* and *bax*, respectively, were increased in the vaginal tissues of women with POP, indicating that apoptotic activity was increased in women with POP. Our previous proteomic analysis [21] demonstrated that many of the differentially expressed proteins in the uterosacral ligaments of POP patients were cytoskeleton proteins and apoptosis-related proteins. Our results confirmed that the uterosacral ligament underwent cytoskeletal changes and apoptosis during the process of POP both in vitro and in vivo.

The most important collagen subtypes in the pelvic floor supportive structures are collagen I and III, which are also the main components of the ECM. Their stability mainly depends on the balance between MMPs and TIMPs. Previous studies [22–25] have detected ECM-related proteins in the vaginal wall and uterosacral ligament tissues of POP and non-POP patients. These studies revealed decreases in the levels of collagen I in the uterosacral ligaments and anterior vaginal walls of POP patients. However, differences in the levels of collagen III, MMP1, 2 and 9, TIMP1 and TIMP2 between POP and non-POP patients were quite controversial [14]. In our study, we found a significant decrease in the expression of collagen I and III and an increase in MMP1 expression which was not statistically significant in cells from POP patients compared to those from non-POP patients, which was consistent with some previous results but inconsistent with others. These controversial results may partially be caused by the approaches used in these studies, including different tissues (the uterosacral ligament or vaginal wall) or cells (primary cultured human fibroblasts derived from the uterosacral ligament, cardinal ligament or vaginal wall), different detection methods (Western blotting, qPCR, Luminex Assay, Masson staining or immunohistochemical staining) and different criteria for patient recruitment (POP-Q stages, pre- or post-menopause, etc.), leading to bias.

We then simulated the development of POP by mechanically stretching hUSLF cells. The cell volume became larger, the long axis became longer, and F-actin stress fibres became thicker after stretching. The composition and metabolic process of the ECM also changed, as collagen I and III were decreased and MMP1 was increased after stretching. In addition, the number of apoptotic cells increased after stretching. Our results were consistent with previous studies. Ewies et al. [26] used cDNA microarrays to detect differences in primary cultured fibroblasts with and without mechanical stretching. They found that the genes involved in actin cytoskeleton remodelling were significantly changed. The percentage of fibroblasts with abnormal F-actin morphology was significantly higher under stretching treatment than in the static state. The cellular changes caused by stretching were similar to those observed in POP patients. Mechanical stimulation can cause hUSLFs to adopt a POP-like state.

Mechanical strain activates multiple signalling pathways, such as the TGF-β/Smad, PI3K/AKT, MAPK and AGE/RAGE pathways, which can transmit mechanical signals and regulate collagen synthesis and metabolism. Vetuschi et al. [27] reported that the expression level of AGE was increased in the vaginal tissues of POP patients. Li et al. [28] found that the PI3K/Akt pathway was activated in the uterosacral ligaments of women with POP.

The MAPK family is a family of serine/threonine protein kinases. There are three main MAPK pathways: the ERK, p38 MAPK and JNK pathways. Vetuschi et al. [27] observed that ERK1/2 and p-ERK were upregulated in the vaginal walls of women with POP compared with the control women. In our study, we found that the p38 MAPK pathway was activated in hUSLFs after mechanical stress, indicating the involvement of the p38 MAPK pathway in POP. Cornelissen et al. [29] demonstrated that pressure dilation of the human saphenous vein leads to the activation of p38, which was also related to cell apoptosis. p38 MAPK was also involved in mechanical transduction in cells, contributing to ECM and cytoskeleton remodelling in tumours, in the cardiovascular system and under other physiological and pathological conditions [30, 31]. However, there were no reports related to p38 MAPK in this pelvic floor disease. In this study, we preliminarily identified a notable change in p38 MAPK in pelvic ligament fibroblasts after mechanical stretching for the first time, providing a new possible mechanism of POP development. However, further investigationis are required to clarify whether the overexpression of these signalling molecules can manipulate the morphology and behaviours of ligament fibroblasts.

## Conclusion

Failure of the pelvic support structures to resist excessive mechanical damage is a potential underlying mechanism of POP development. In our study, we demonstrated that mechanical stretching can induce ligament fibroblasts to undergo POP-like changes and that the p38 MAPK pathway may participate in this process. Given the mechanical sensitivity and mechanical reactivity of pelvic ligament fibroblasts, it is possible to adjust the actions of these cells by modulating specific mechanical signal molecules, providing new prevention or therapeutic strategies for this disease.
